# New Strategy of Reducing Biofilm Forming Bacteria in Oral Cavity by Bismuth Nanoparticles

**DOI:** 10.1155/2021/6695692

**Published:** 2021-02-02

**Authors:** Sahar Rostamifar, Azita Azad, Ali Bazrafkan, Farzan Modaresi, Shekoufeh Atashpour, Zahra Kargar Jahromi

**Affiliations:** ^1^Student Research Committee, School of Dentistry, Shiraz University of Medical Sciences, Shiraz, Iran; ^2^Oral and Dental Disease Research Center, Department of Oral & Maxillofacial Medicine, School of Dentistry, Shiraz University of Medical Sciences, Shiraz, Iran; ^3^Departments of Microbiology, Advanced Medical Sciences and Technology, and Central Laboratory Research, Jahrom University of Medical Sciences, Jahrom, Iran; ^4^Departments of Pharmacology, Advanced Medical Sciences and Technology, and Central Laboratory Research, Jahrom University of Medical Sciences, Jahrom, Iran; ^5^Central Research Laboratory, Jahrom University of Medical Sciences, Jahrom, Iran

## Abstract

**Objective:**

*Enterococcus faecalis* and *Streptococcus salivarius* are the most important species in dental decay and producing biofilm. Treatment with chlorhexidine 2% mouthwash for 7 days is the best way to eliminate these bacteria. However, due to the ability of these bacteria to survive in harsh environments, increasing emergence of bacterial resistance against available antibiotics, and favorable properties of nanoparticles including broad spectrum antimicrobial activity and lower toxicity, we decided to evaluate reducing biofilm forming bacteria in oral cavity by bismuth nanoparticles.

**Materials and Methods:**

This was a cross-sectional study of 40 samples isolated from the patients visiting dental clinics in Shiraz in 2019. Samples, which showed growth, were cultured on blood agar plates and incubated for the PCR procedure. Nanoparticle powder was dissolved in high-purity water, and the final concentration of bismuth nanoparticles (BiNPs) was measured with a spectrophotometer. Minimum inhibitory concentration (MIC) of BiNPs against *E. faecalis* and *S. salivarius* was determined by the microbroth dilution method according to methods for antimicrobial susceptibility tests. Also, bactericidal assays were conducted in a Mueller-Hinton broth medium and reported as the concentration of BiNPs that reduced the viable bacterial count by 99.9%. Statistical analysis was carried out using SPSS 21 and one-way analysis of variance, and *P* values less than 0.05 were considered significant.

**Results:**

MICs of BiNP suspension against *Streptococcus salivarius* and *Enterococcus faecalis* were 2.5 and 5 *μ*g/ml, respectively. Minimum bactericidal concentrations (MBC) of BiNP suspension against *Streptococcus salivarius* and *Enterococcus faecalis* were 5 and 10 *μ*g/ml, respectively. Antibacterial activity of BiNPs was compared with chlorhexidine 2%. MICs of BiNPs against *Streptococcus salivarius* and *Enterococcus faecalis* were one-twentieth less than those of chlorhexidine. MBC of BiNPs against both pathogens was one-tenth less than those of chlorhexidine.

**Conclusion:**

BiNPs were more effective than chlorhexidine, and MIC and MBC of bismuth nanoparticles are lower than those of chlorhexidine.

## 1. Introduction

The mouth contains around 500 different types of microbes, some of which cause infectious diseases [1]. Any change or disturbance in oral microflora causes oral infection. Tooth decay, periodontal diseases, dentoalveolar infections, and oral mucosal infections are the most important oral infectious diseases [2, 3]. Common oral microflora are *Streptococcus*, *Staphylococcus*, *Lactobacillus*, *Veillonella*, and *Neisseria*. Of these, *Streptococcus* is the first organism that is detected in the mouth of newborn infants, especially the *salivarius*, *oralis*, and *mitis* species.

*Streptococcus* is the most prevalent bacteria in the oral cavity. It is facultatively anaerobic, catalase-negative, and gram-positive cocci. Oral *streptococci* are divided into five different groups of *mutans*, *salivarius*, *anginosus*, *oralis*, and *mitis* [2, 3]. Sundqvist assessed the significance of anaerobic bacteria in endodontic infections in 1976 and showed that all samples of teeth with periapical lesions were positive for bacterial infection except one. This study also showed that rod-shaped black-pigmented anaerobic bacteria were isolated from painful teeth [4].

*Enterococcus faecalis* is a gram-positive facultative anaerobic bacterium that causes 80-90% of enterococcal infections in humans, and most cases were isolated from failed root canals [5]. Sundqvist [6] isolated *Enterococcus faecalis* (EF) from 38% of teeth with failed root canal treatment. Only 33% of the teeth with EF were successfully treated after root canal refilling [6]. Although molecular studies showed that *Enterococcus faecalis* is not the prevalent cause of endodontic infections, it is the most common cause of refractory and secondary endodontic infections [7].

Chlorhexidine gluconate is a bisbiguanide compound with broad-spectrum antimicrobial activity and low toxicity and is the most effective chemical antibacterial mouthwash authorized by the Food and Drug Administration and the American Dental Association. It inhibits smooth surface decay, controls dental gingivitis, disinfects hands and dentures, and reduces microbial plaque. Therefore, it is recognized as a gold standard to control microbial plaque. Administration of chlorhexidine 2% twice a day completely removes microbial plaque [8].

Many studies have been directed towards finding an alternative strategy for prevention or eradication of *E. faecalis* and *S. salivarius* from the root canal system [9–12]. Amongst these strategies, nanoparticles, typically 0.2–100 nm in size, showed good results as novel antimicrobial agents. Nanotechnology is used in various fields including medical diagnostics, food, medicine, chemistry, biotechnology, environment, and physical energy. It is regarded as an interdisciplinary technology [13, 14]. Up to now, the metals most frequently used for biomedical applications include gold, titanium, silver, copper, zinc, magnesium, and bismuth [14]. Bismuth nanoparticles are found as bismuthinite (bismuth sulfide), bismite (bismuth oxide), and bismutite (bismuth carbonate) [15]. Bismuth compounds are mainly used for treatment of Helicobacter pylori-induced peptic ulcer [15, 16]. Furthermore, bismuth nanoparticles are currently used to detect bacterial resistance to antibiotics [17]. One of the characteristics of this nanoparticle is prevention of biofilm formation in *Streptococcus mutans*, which is the main cause of tooth decay [18, 19]. Hernandez-Delgadillo et al. showed that a combination of bismuth nanoparticles with minerals inhibited growth of *Enterococcus faecalis*, *Escherichia coli*, and *Candida albicans*. It also destructed biofilms of *Enterococcus faecalis* 24 hours after exposure [20]. Therefore, this study assessed the effect of bismuth nanoparticles on *Streptococcus salivarius* and *Enterococcus faecalis*, which are important causes of tooth decay and biofilm, and the effect of bismuth nanoparticles using MIC and MBC was compared with that of chlorhexidine regarding standard strains.

## 2. Materials and Methods

### 2.1. Sampling

Forty patients, ages from 18 to 45 years old, including 22 males and 18 females, referred to the Endodontic Ward of Shiraz University of Medical Science for endodontic pretreatment, provided root canal samples, which were then analyzed for the presence of *E. faecalis* and *S. salivarius*. All samples were obtained from patients who had rooted canal therapy completed more than 1 year ago [21]. Patients who were pregnant, diabetic, and smokers and those requiring pretreatment due to missing canals, broken instruments, perforations, ledges, or calcified root canals were excluded. None of the selected teeth have termini of the root canal filling more than 5 mm short in radiographic findings and periodontal pockets deeper than 4 mm. After supragingival scaling and isolation with a rubber dam, samples were taken by one of the authors as previously described by Gomes et al. [22]. The teeth and the adjacent field were decontaminated with a 2.5% sodium hypochlorite for 30 s each and then inactivated with 5% sodium thiosulfate. As the previous restorations were removed and the access cavities prepared, the pulp chambers were disinfected with 5.25% sodium hypochlorite, and the obturation materials were removed with ProTaper nickel-titanium rotary instruments SX-F2 (WNT, India) under irrigation with sterile saline. The microbial samples were collected by inserting two sterile paper points into the working length of the canal and keeping them in place for 60 s. The debris on the paper points were transferred into sterile 2 ml Eppendorf tubes containing viability medium Gotenberg agar III transport medium and evaluated immediately within 2 hrs. After shaking the samples in a mixer for 60 s (Vortex, Scientific Industries Inc., Springfield, MA), 1 ml of each sample was used for culture, and the other 1 ml was frozen at −20°C for by PCR procedures [22]. The study was approved by the Ethics Committee of Shiraz University of Medical Science IR.sums.dental.rec.1399.119). Patients were informed of the study procedures and goals, and written consent was obtained [21].

### 2.2. Preparation of Standard McFarland 0.5 Solution

To prepare the standard McFarland 0.5 solution, 0.5 ml of BaCl_2_ (0.048 mol/l) (2H_2_O *W*/*V* BaCl_20_ 1/175%) was added to 99.5 ml of sulfuric acid (0.18 mol/l) (*V*/*V* 1%). The suspension was stirred continuously, and the standard optical density was determined by absorbance measurement using a spectrophotometer at an optical length of 1 cm. The absorbance of 625 nm should be between 0.8 and 0.13 [23].

### 2.3. Culturing Reference Strains

Standard strains of *Enterococcus faecalis* (ATCC 51299) and *Streptococcus salivarius* (ATCC 13419) obtained from the American Type Culture Collection were studied. The syringe containing the standard bacteria was sterilized with alcohol. It was cracked, and 2 cc of typical broth medium was added to the syringe and mixed. The mixture was divided into several plates containing blood agar medium. The plates were incubated at 37°C [21].

### 2.4. Preparation of 0.5 McFarland Solution of Studied Bacteria

Several colonies of bacteria were dissolved in 1 cc of physiological serum. Turbidity of bacterial solution was compared with the standard 0.5 McFarland solution [21].

### 2.5. Preparation of Bismuth Nanoparticle Suspension

Eight milligrams of nanoparticle powder (Nano Scientific Co., USA) was dissolved in 200 ml of high-purity water and sonicated (Biometra, Germany) for 20 minutes at 900 watts. The sonicated solution was sterilized by passing through a 0.2 *μ*m filter (Control Biogene, Spain), and the final concentration of BiNPs was measured with a spectrophotometer (Eppendrof, Germany). The shape, size, and distribution of BiNPs have been characterized by the high-resolution transmission electron microscopy (TEM) with a JEM-1011 microscope (JEM-1011 “JEOL Ltd.,” Japan) [21].

### 2.6. MIC and MBC Calculation

Minimum inhibitory concentration (MIC) of BiNPs against *E. faecalis* and *S. salivarius* was determined by the microbroth dilution method according to antimicrobial susceptibility tests for bacteria that grow aerobically, 11th edition. Twelve tubes were selected and coded. One milliliter of Müller-Hinton broth medium was added to tubes 1-10. BiNP suspension (1280 *μ*l or 0.1 mg/ml) was poured into another test tube. Müller-Hinton Broth medium (720 *μ*l) was added to the tube. The medium was prepared at a concentration 2.5 times greater than the original concentration. After the vortex, 1 ml of nanoparticle suspension containing 64 *μ*g of nanoparticle was added to tube number 1. Final concentration of tube number 1 would be 32 *μ*g. The contents of tube number 1 were vortexed, and one milliliter of tube number 1 was added to tube number 2. After the vortex, one milliliter of tube number 2 was added to tube number 3. The procedure was continued to tube number 10. In the next step, 1 ml of microbial suspension (1 × 10^6^) was added to each tube. The tubes were incubated at 37°C for 24 h. The lowest concentration that inhibited bacterial growth was MIC. To determine MBC, 100 *μ*l of diluted solution with no sign of turbidity (prior to addition of microbial suspension) was cultured on Müller-Hinton agar under aseptic conditions. The suspension was incubated at 37°C for 24 h. The colonies were counted, and the lowest concentration that destroyed 99.9% of isolates was determined as MBC [21].

### 2.7. DNA Extraction

Bacteria were cultured on nutrient agar medium. After securing a single colony, 2-3 colonies of bacteria were removed using a loop under sterile conditions. The colonies were then sterilized in a microtubule containing 200 *μ*l of distilled water. DNA was extracted using a QIAGEN DNA extraction kit according to its protocol. To ensure the required purity, its absorption was determined by spectrophotometry at 260 nm.

### 2.8. Bacterial Detection by PCR Method

The PCR method and the 16S rRNA gene were used for bacterial strain detection. First, Mastermix QIAGEN was mixed with a suitable amount of sterile water, and the desired primers were added to the mixture. Mastermix was then divided into 0.2 microtubes, and DNA was added to the tubes. It was transferred to an Eppendorf thermocycler to carry out the PCR program for *S. salivarius* and *E. faecalis* (initial denaturation at 94°C for 5 minutes, 30 cycles of denaturation at 94°C for 45 seconds, annealing (annealing temperature: 60°C) for 45 seconds, extension (temperature: 72°C) for 45 seconds, and post extension for 10 minutes) [21]. Electrophoresis was carried out following PCR, and 310 bp bands were identified as *E. faecalis* and 544 bp bands as *S. salivarius*. The sequence of primers used in this study was [21] the following: *S. salivarius* forward: GTGTTGCCACATCTTCACTCGCTTCGG, and *S. salivarius* reverse: CGTTGATGTGCTTGAAAGGGCACCATT; *E. faecalis* forward: GTT TAT GCC GCA TGG CAT AAG AG, and *E. faecalis* reverse: CCG TCA GGG GAC GTT CAG.

### 2.9. Statistical Analysis

Statistical analysis was carried out using SPSS 21 and one-way analysis of variance (ANOVA) followed by Duncan post hoc test. A statistical *P* value less than 0.05 was considered significant.

## 3. Results

### 3.1. Identification of *E. faecalis* and *S. salivarius* by PCR Procedure

**Figure 1:**
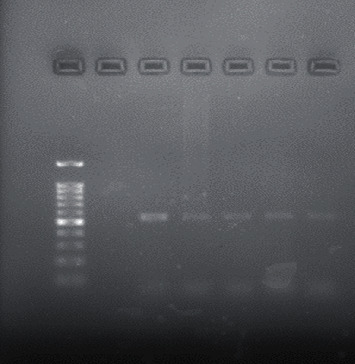
Electrophoresis of *S. salivarius* 16S rRNA gene on 1% agarose gel. The target gene was of 544 bp. From left to right: ladder 100 bp, negative control, positive control (544 bp), and positive samples.

**Figure 2:**
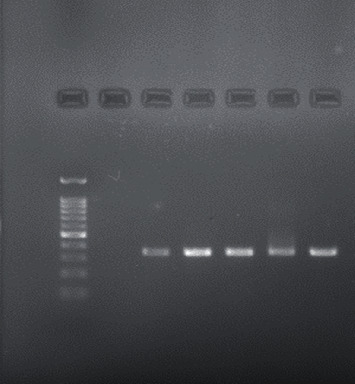
Electrophoresis of *E. faecalis* 16S rRNA gene on 1% agarose gel. The target gene was of 310 bp. From left to right: ladder 100 bp, negative control, positive control (310 bp), and positive samples.

Forty adult patients consisted of 22 men and 18 women with a mean age of 30.175 (range from 18 to 45 years) provided samples in the study. Twenty-five out of 40 samples (62.5%) revealed the presence of *E. faecalis* and eighteen out of 40 samples (45%) revealed the presence of *S. salivarius* by PCR identification technique loaded into 1% agarose electrophoresis gel (Figures 1 and 2). However, seven root canal samples (17.5%) showed no growth of both bacteria. Details regarding subjects are presented in Table 1.

### 3.2. BiNP Characterization

**Figure 3:**
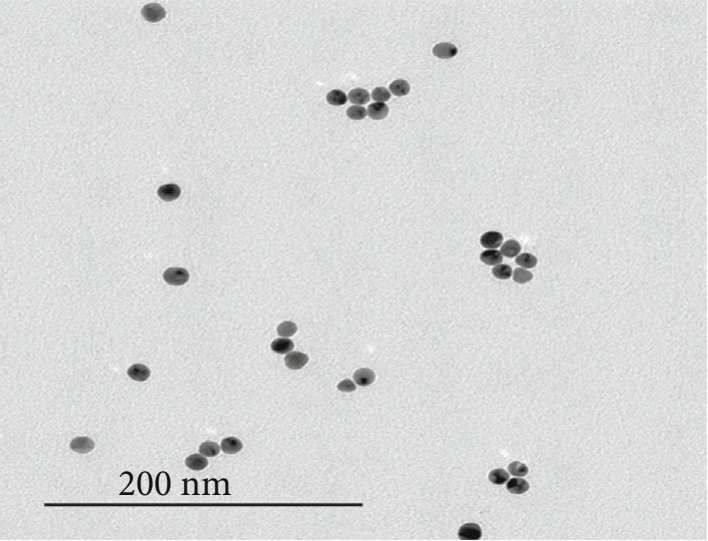
Bismuth nanoparticle electron microscope image diameter and size statistics: JEOL JEM-1011 Transmission Electron Microscope. Mass concentration: Freeze Dryer Christ Alpha 1-4 LSC. Zeta potential: Malvern Zetasizer Nano ZS9. UV-vis: Spectrophotometer NanoDrop 2000c.

BiNPs have been used with the aim of estimating their antimicrobial activity against *E. faecalis* and *S. salivarius*. By transmission electron microscopy, it has been found that nanoparticles have an average particles size of 40 nm with a spherical form (Figure 3).

### 3.3. Antimicrobial Susceptibility Testing

**Figure 4:**
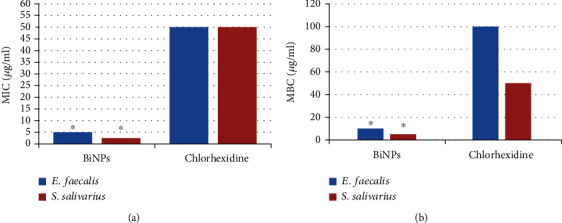
Comparison of MIC (a) and MBC (b) of chlorhexidine and bismuth nanoparticles in *E. faecalis* and *S. salivarius*. ^∗^Significant difference between BiNPs and chlorhexidine groups.

To explore the possible antibacterial activity of bismuth nanoparticles, their effects on *E. faecalis* and *S. salivarius* growth were determined. The results showed *E. faecalis* and *S. salivarius* growth inhibition at concentrations ranging from 0.625 *μ*g/ml to 20 *μ*g/ml (geometric mean: 2.337 *μ*g/ml). Also, the MBC values were between 1.25 *μ*g/ml and 40 *μ*g/ml (geometric mean: 4.781 *μ*g/ml).  Table 2 and Figure 4 show the MIC and MBC values of all tested isolates towards BiNPs. MIC and MBC of *E. faecalis* isolates were calculated, and MIC ranged from 2.5 to 5 and MBC ranged from 5 to 20 *μ*g/l. MIC was 5 in most samples, and MBC was 10 *μ*g in most isolates. MIC and MBC were also assessed in *S. salivarius* isolates, and MIC ranged from 1.5 to 25 and MBC ranged from 2.5 to 5 *μ*g/l. MIC in most samples was 2.5, and MBC in most isolates was 10 *μ*g. The results of this study showed stronger antimicrobial activity of bismuth nanoparticles against both clinical isolates and standard strains of *S. salivarius* and *E. faecalis*. This was while the MIC and MBC values of chlorhexidine were recorded as 50 *μ*g/ml and 100 *μ*g/ml. Therefore, MIC and MBC of bismuth nanoparticles were lower than chlorhexidine in both bacteria, which indicated that bismuth nanoparticles were more effective than chlorhexidine.

## 4. Discussion

*E. faecalis* and *S. salivarius* are the most important species in dental decay, biofilm formation, and endodontic infection. In this study, the prevalence of *E. faecalis and S. salivarius* among the selected patients was 62.5% and 45%, respectively. A study by Rôças and colleague showed that *E. faecalis* was detected in 66.6% of cases of persistent endodontic infections associated with root-filled teeth and much more likely to be seen in cases of failed endodontic therapy than in primary infections [24]. Furthermore, previous researches demonstrated that *E. faecalis* was the most prevalent species recognized by PCR in teeth with failing endodontic treatment and ranged between 24 and 77% which can be due to the differences in the methods of identification [25, 26]. Another study concluded that microbial flora in root-filled teeth were predominantly facultative anaerobes including *S. salivarius* and gram-positive species as *E. faecalis* was the most common isolated species [27].

Chlorhexidine is widely used as a gold standard for removal of microbial plaque. It is a diguanide hexidine with potent antiseptic properties. Despite all positive properties of the compound, its long-term use increases the risk of oropharyngeal cancer due to the tooth and tongue staining, altered taste, and high alcohol level (12%) [28, 29]. Furthermore, appropriate antibiotic therapy is diminishing day after day due to increased bacterial resistance to antibiotics and has encouraged development of alternative therapeutic strategies. Amongst these strategies, nanomaterials have turned up as notable and novel antimicrobial agents [30]. Various studies have shown that nanoparticles are effective in a wide range of fungi, protozoa, and even viruses unlike conventional antibiotics that only kill bacteria [31–36]. Antimicrobial activity of bismuth nanoparticles was assessed against two dental plaque infected with *Streptococcus salivarius* and *Enterococcus faecalis*. The microdilution broth method was used in this study, which is used as a standard laboratory method with more accuracy and reliability and ease of interpretation than other laboratory methods (e.g., the well diffusion method) [36, 37]. The results of this study showed that BiNPs had an antimicrobial effect on *E. faecalis* and *S. salivarius* with MIC of 5 and 2.5 *μ*g/ml, respectively. Furthermore, this study indicated that bismuth nanoparticles had lower MIC and MBC than chlorhexidine, so BiNPs have stronger antibacterial activity than chlorhexidine.

Given the benefits of nanoparticles, various studies have assessed the effect of nanoparticles on bacteria that cause tooth decay, especially silver nanoparticles. Sadeghi et al. compared the antimicrobial activity of silver nanoparticles with chlorhexidine against *Streptococcus sanguis* and *Actinomyces viscosus* and showed stronger antimicrobial activity of silver nanoparticles than chlorhexidine [37]. Bismuth nanoparticles also showed the same effect as silver nanoparticles against *E. faecalis* and *S. salivarius* in this study. Given the advantages of bismuth nanoparticles, they are superior to silver nanoparticles. Niakan et al. compared the antimicrobial activity of silver nanoparticles and deconex disinfectant against *Staphylococcus aureus* and *Pseudomonas aeruginosa* and showed the stronger antimicrobial activity of silver nanoparticles [38]. Bismuth nanoparticles also showed antimicrobial properties in concentrations less than 1 mM and could inhibit bacterial growth. Hernandez-Delgadillo et al. also showed that colloidal bismuth nanoparticles also inhibit the growth of *Streptococcus mutans* by 69% and prevent biofilm formation by 100% [17]. These nanoparticles could also prevent biofilm formation in *Streptococcus mutans* [19].

These nanoparticles inhibited the growth of *S. salivarius* and *E. faecalis* in this study. MICs against *S. salivarius* and *E. faecalis* were 2.5 and 5 *μ*g/ml, respectively, and MBCs against these two bacteria were 5 and 10 *μ*g/ml, respectively. *S. salivarius* seems to be more sensitive to bismuth nanoparticles than *E. faecalis*. Antibacterial activity of bismuth nanoparticles was compared with 12% chlorhexidine in this study. MICs of these particles against *S. salivarius* and *E. faecalis* were one-twentieth and one-tenth to one-twentieth less than those of chlorhexidine (MIC: 50 *μ*g/ml). MBCs of these particles against these two microorganisms were one-tenth less than those of chlorhexidine (MBC in *S. salivarius*: 50 *μ*g/ml, and in *E. faecalis*: 100 *μ*g/ml). However, studies have shown that chlorhexidine is stronger than other antibacterial products in the removal of dental plaques infected with microorganisms [39–41]. Haffajee et al. showed that chlorhexidine mouthwash had stronger antimicrobial activity than herbal mouthwash using the serial dilution method and MIC calculation [42]. Moeintaghavi et al. compared MICs of chlorhexidine mouthwash with Persica herbal mouthwash against *Streptococcus sanguis* and *Actinomyces viscosus* and showed that chlorhexidine was stronger than Persica [43]. Silver nanoparticles have multiple purposes, but bismuth nanoparticles are less toxic and more efficacious with fewer side effects [44].

Given the positive results and advantages of nanoparticles, there is still no adequate number of studies to address the long-term effects and toxicity of these particles on living organisms, including humans. Further studies are needed to assess antibacterial activity of these particles in the in vitro condition. Silver particles seem to deposit in living tissues; however, silver nanoparticles do not seem to accumulate in living organisms and are currently used in different studies. Nanoparticle compatibility and recycling should also be addressed in future studies [45].

Due to the high side effect of routine drugs used for oral infection, low efficacy, drug resistance, and use as mouthwash which could cause sensitivity and have low efficacy, emergence of a new strategy for the treatment of dental caries, oral infection, and infection related to the oral cavity such as endocarditis and septicemia is very critical and essential. In this regard, we used bismuth nanoparticles for overcoming this problem. According to results of the current research, low MIC of BiNPs, high efficacy, and low price, it could be used as an alternative drug or mouthwash for oral infection [10, 21].

## 5. Conclusion and Recommendations

Bismuth nanoparticles could be an interesting alternative to combat *S. salivarius* and *E. faecalis*, which has higher antibacterial activity and lower side effects compared to chlorhexidine and can be suggested to be used in different fields of dentistry. However, bismuth nanoparticles should be addressed in extensive studies. Further studies with a larger sample size should be carried out in this regard. Toxicity and short-term and long-term effects of these nanoparticle in living cells are the most important issues that should be addressed in future studies.

## Figures and Tables

**Table 1:** Presence of *E. faecalis* and *S. salivarius* in samples collected from patients.

Number of bacteria	*S. salivarius*	*E. faecalis*
8	+	−
15	−	+
10	+	+
7	−	−
Total	40	

**Table 2:** MIC and MBC values of bismuth nanoparticles against *S. salivarius* and *E. faecalis*.

Patient	*S. salivarius*		*E. faecalis*	
	The MIC of BiNP (*μ*g/ml)	MBC of BiNP	The MIC of BiNP (*μ*g/ml)	MBC of BiNP
1			5	10
2	2.5	5		
3				
4	2.5	5	5	10
5			2.5	5
6	2.5	5	5	10
7	5	10		
8				
9	2.5	10		
10			2.5	5
11	5	10	10	20
12			5	10
13			5	10
14	5	10		
15				
16	5	10	5	10
17			5	10
18	2.5	5	5	10
19			10	20
20	1.25	2.5		
21				
22			0	10
23	5	10		
24			2.5	0
25	5	10	5	10
26			2.5	0
27	2.5	5		
28				
29			5	10
30	2.5	5	5	10
31			2.5	5
32	5	10	10	20
33				
34			2.5	5
35	2.5	5	0	10
36	2.5	10	0	20
37			2.5	10
38	2.5	5		
39				
40			0	10

## Data Availability

The experimental and clinical data used to support the findings of this study are included within the article.
